# Comparative analysis of postoperative mechanical behavior of immature premolars: apexification vs regeneration

**DOI:** 10.1007/s10266-025-01294-z

**Published:** 2026-03-17

**Authors:** Ana Carolina da Silva, Fernando César de Barros Faria Júnior, Gustavo Guimarães Guerrero, Alexandre Luiz Souto Borges, Marcia Carneiro Valera

**Affiliations:** 1 Department of Dental Materials and Prosthodontics, Institute of Science and Technology, Sao Paulo State University (UNESP), Engenheiro Francisco Jose Longo Avenue, 777 - Sao Jose Dos Campos, Sao Paulo, 12245-000 Brazil; 2 Department of Restorative Dentistry, Endodontic Division, Institute of Science and Technology, São Paulo State University - UNESP, São José Dos Campos, Sao Paulo, Brazil

**Keywords:** Regenerative endodontics, MTA, Apexification, Root canal treatment, Finite element analysis

## Abstract

To compare the mechanical behavior of lower premolars with incomplete root formation treated by apexification and regeneration and assess revascularization's impact on dentin thickness using finite element analysis. A mandibular bone model was obtained from a computed tomography scan and modeled using the CAD Rhinoceros 7.0 software program. The models were composed of enamel, dentin, pulp, periodontal ligament, cortical bone, medullary bone, gutta-percha, MTA, blood clot, and composite resin. The study groups were: IT (Intact Tooth); AC (Accessed Tooth); R (revascularization); and AX (apexification). After modeling the structures, the geometries were exported to the Ansys 13.0 finite element analysis software program. The models were submitted to loads of 200 N on the distal sulcus of the occlusal surface. The loading was directed at 45° to the occlusal plane. The results were calculated and qualitatively analyzed using displacement maps and maximum principal stress. The apexification models supported lower compressive and tensile stresses when compared to the revascularization models. Revascularization reduced stress concentration in the periradicular tissues, whereas apexification resulted in lower tensile stress in coronal dentin.

## Introduction

Endodontic treatment in immature and necrotic roots has been frequently studied over the years due to the numerous challenges it presents. The apex with incomplete formation is considered one of the most relevant complexities of endodontic treatment since the lack of apical constriction favors the extrusion of filling materials during obturation, regardless of the technique used [1]. To solve this problem, the apexification process with mineral trioxide aggregate (MTA) was implemented because it presents several advantages such as biocompatibility, the ability to form a mineralized barrier and apical sealing, as well as acting as a tissue repair stimulator [2–4].

According to studies by Shabahang et al. [5], MTA application in dog teeth with incomplete root formation enables the formation of an apical hard tissue barrier. In addition, another study has shown that MTA has good sealing capacity and induces less stress in radicular dentin [6]. However, this material is difficult to handle, promotes tooth discoloration, and does not increase the root wall thickness [1, 6, 7].

Another alternative for endodontic treatment of teeth with immature roots and pulpal necrosis is the pulp revascularization procedure. This technique consists of inducing periapical bleeding, which fills the root canal with stem cells, promotes cementoblastic activity, and subsequently forms mineralized tissue in the dentin walls [8]. Studies in the literature show that this technique promotes a high success rate, which is equivalent to the apexification treatment [9–12]. However, few studies in the literature have been conducted on the mechanical strength of immature teeth submitted to the pulp revascularization process.

Computational tools have been developed to solve engineering problems. The FEA method has been frequently applied for experimental tests in the dentistry field. This analysis enables modeling non-homogeneous structures with complex geometries, as well as irregular structures of natural and artificial tissues [13]. This method allows easy obtainment of a 3D mathematical model, simulates load application without damaging the tissues, and its results are simple to interpret [14]. It is possible to verify locations that are more susceptible to failures using FEA, as it can highlight the stress concentration locations and the critical points with the greatest overload [15]. Thus, the obtained three-dimensional model provides greater information, as it reproduces the anatomical structures and the application of forces in a simplified way, which makes the mathematical simulation closer to reality [16]. In turn, an FEA analysis can be applied to evaluate the mechanical resistance of apexified and revascularized immature teeth.

Within this perspective, this study evaluated the mechanical behavior of lower premolars with immature roots after implementing the apexification and revascularization techniques through the FEA method. The tested hypothesis was that the pulp revascularization technique would show better mechanical behavior in stress distribution when compared to the apexification technique.

## Materials and methods

### Three-dimensional modeling

The FEA models were validated by an indirect method [17]. The geometric measurements and proportions to elaborate the first mandibular premolars with incomplete root formation (and single canal anatomy) in 3D models were obtained by applying reverse engineering techniques. A lower immature premolar was scanned using a cone beam computed tomography (Skyscan 1172, Micro Photonics Inc., Aartselaar, Belgium). X-ray projections were reconstructed into 3D models and stored as a sequence of bitmap (BMP) files. The premolar models were made following the Bio-CAD protocol [16], in which the STL (Standard Tessellation Language) format is used to create a solid model of the analyzed region.

The 3D solid models were created using the CAD Rhinoceros 7.0® software program (McNeel North America, Seattle, WA), which uses NURBS (Non-Uniform Rational Bézier Spline) modeling. The geometric construction simulated the conditions used to carry out an experimental test in which the 3D models consisted of enamel, dentin, pulp, periodontal ligament, cortical bone, and medullary bone. A perfectly bonded contact simplification hypothesis (coincident nodes) was established.

All materials used in the study groups were created in a 3D model by FEA under the same dimensions as the real model and in the same program. With the intact models finalized, the geometry was exported to the Ansys® finite element analysis software program (version 13.0; Ansys, Canonsburg, PA) in STEP 214 Automotive Design (STP) format and subsequently imported into CAE (Computer Aided Engineering) from Ansys® (version 13.0; Ansys, Canonsburg, PA). Then, the meshes were made and adjusted to obtain sufficiently accurate results until a convergence of 10% was obtained.

**Fig. 1:**
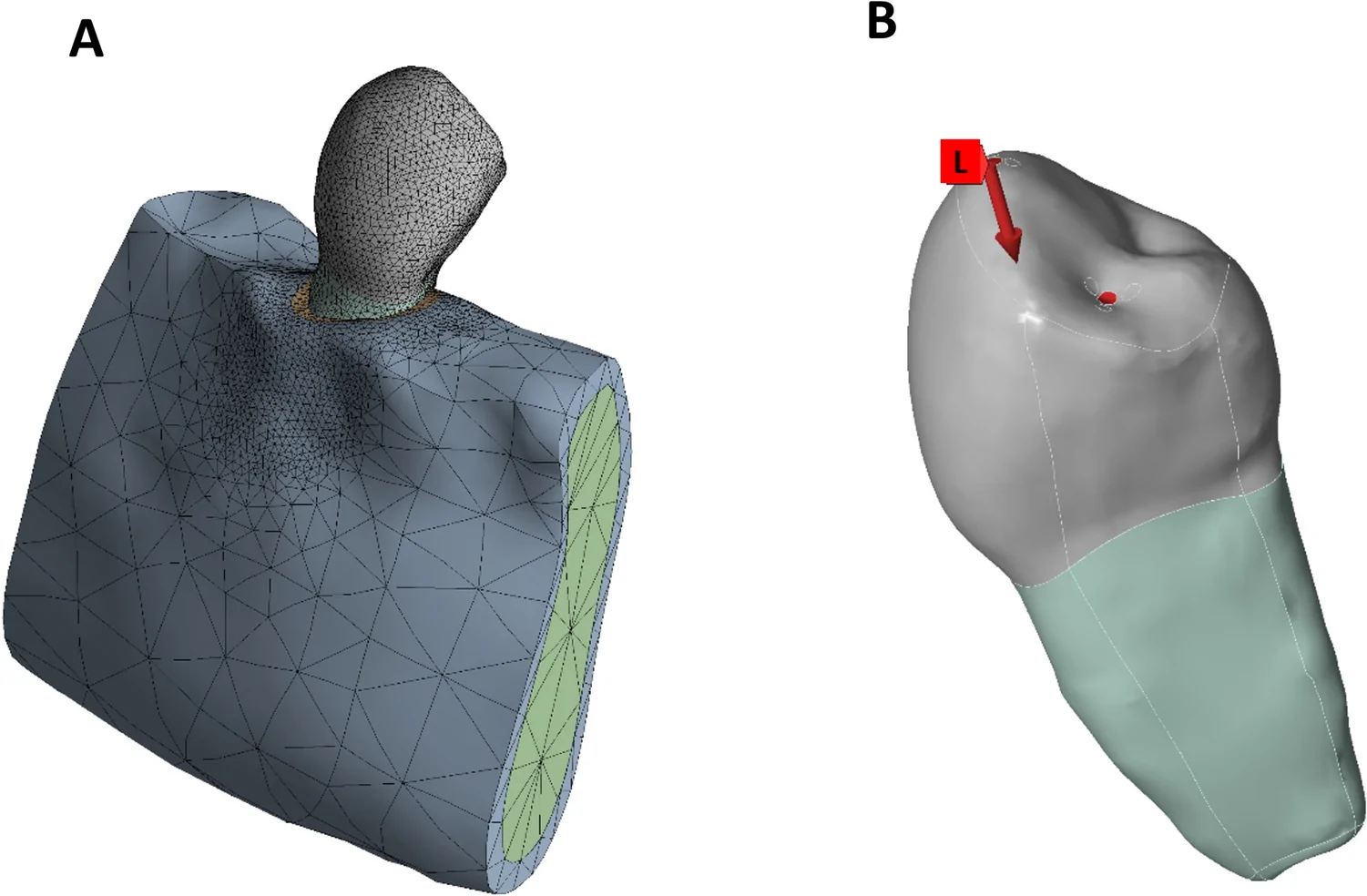
Three-dimensional model of the sagittal section of the mandible with lower first premolar. Legend: The blue color in the image represents the cortical bone, while the green color represents the medullary bone of the mandible. **A** Presence of mesh nodes of a geometric structure; **B** finished structure; **C** and **D** the red arrow and letter “L” indicate the load application region

A controlled mesh defined by quadratic tetrahedral elements characterized by a pyramid with a triangular base was used, with a node at each vertex and another at the center of each edge, totaling 10 nodes per element [18]. The total number of tetrahedron elements the control group was 147,037 with 228,255 nodes, group AX 242,570 nodes and 144,351 elements, group R 247,330 nodes and 152,637 elements and group AC 213,828 nodes and 135,855 elements. All meshes showed an average of 0.55 in the maximal skewness, 0.99 in the element quality and 4.99 in the aspect ratio (Fig. 1).

**Fig. 2:**
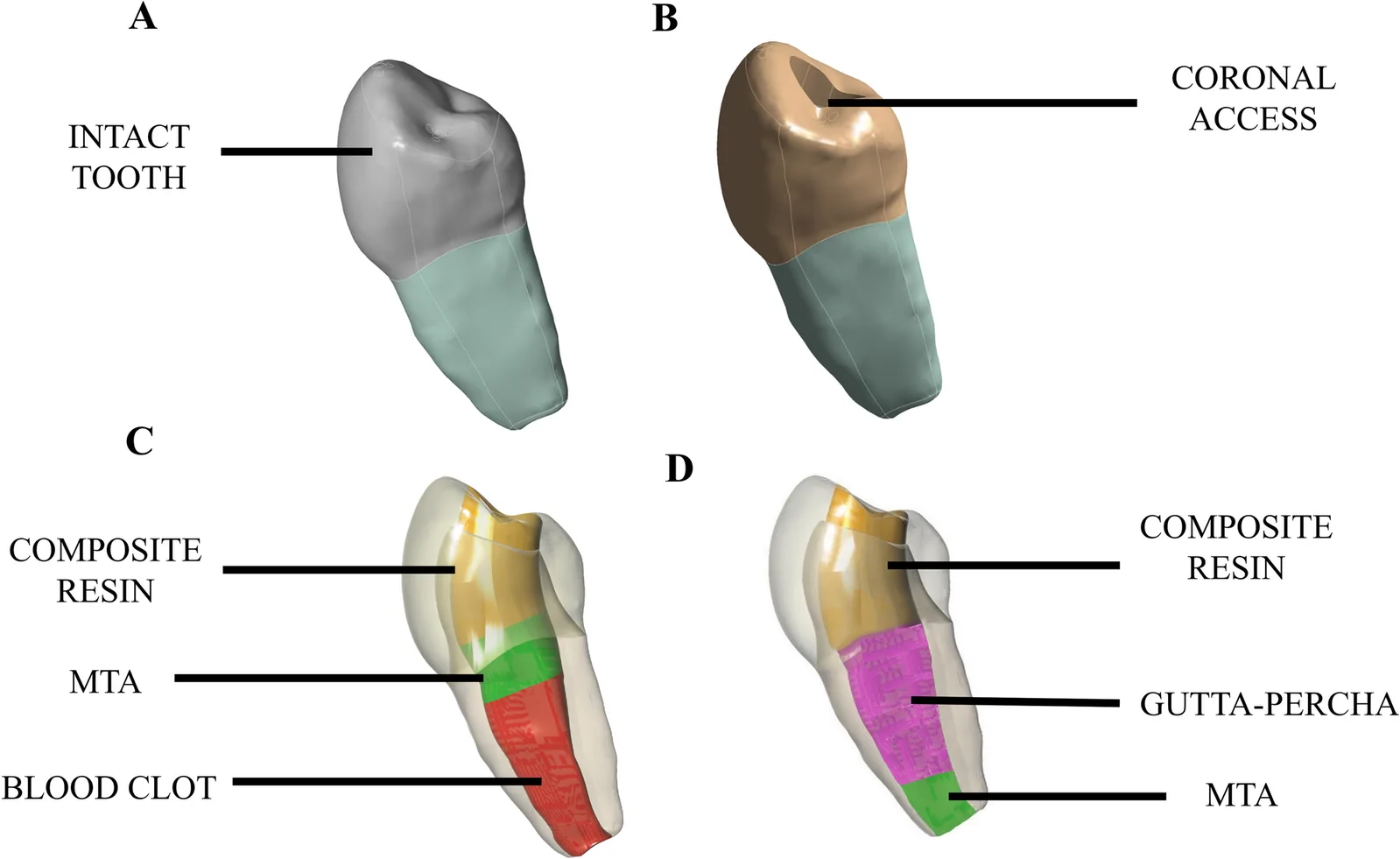
3D models corresponded to: **A** IT (intact tooth); **B** AC (accessed tooth); **C** R (revascularization); and D) AX (apexification) with the respective intraradicular materials

The interface between the different model parts was represented most simply by coincident nodes, and the contact relevance between all geometries was 50%. Two lower premolars were designed (with and without an access cavity) to form the groups. The endodontic techniques within the coronal access model were simulated to: revascularization (R—5 mm of MTA in the cervical region and coronal sealing with composite resin); apexification (AX—5 mm of MTA in the apical region, gutta-percha, and coronal sealing with composite resin) [19], and accessed tooth (AC), while the lower premolar without access cavity corresponding to an intact tooth (IT) (Fig. 2).

### Finite element analysis

The model obtained was fixed based on the bone structures around the tooth, and a load of 200 N directed at 45° to the occlusal plane was applied in the distal sulcus of the occlusal surface [20, 21]. The selected load of 200 N was chosen for the premolar loading, considering both experimental bite-force literature [22, 23] and precedent in the finite element literature [14, 24].

To avoid numerical singularities, the load was distributed uniformly over a small circular contact area (diameter = 1.0 mm). The tooth, PDL, and alveolar bone were modeled as bonded (tie) contacts to ensure force transmission. Boundary conditions were applied by fully constraining (UX = UY = UZ = 0) all nodes on the inferior surface of the alveolar bone. Then, mesh convergence was assessed (refinement levels n1–n3).

All elements were considered isotropic, linear, and elastic. Elastic, linear, and homogeneous properties of teeth were assumed for enamel, dentin [25], pulp [26], cementum [27], cortical bone, medullary bone [25], gutta-percha, MTA, and composite resin [6] (Table 1).

**Table 1:** Mechanical properties of the materials used in the study

Materials	Young’s modulus (MPa)	Poisson coefficient
Enamel [22]	84,100	0.20
Dentin [22]	18,600	0.31
Cementum [24]	3,730	0.3
Cortical bone [22]	15,000	0.30
Medullary bone [22]	1500	0.30
Pulp [23]	2	0.45
Gutta-percha [6]	140	0.45
MTA [6]	11,760	0.314
Composite resin [6]	16,400	0.28
Periodontal ligament (PDL) [22]	70.3	0.45

The processing was subsequently performed by the Ansys® finite element program through the Mechanical APDL solver, in which the stress distribution and coherence in the results were verified. The failure criterion adopted considered the mechanical characteristics of each tissue. For brittle materials such as dentin and enamel, as well as for the periodontal ligament (PDL), the Maximum Principal Stress was used to identify regions subjected to tensile loads. For the alveolar bone, the Minimum Principal Stress was applied to evaluate compressive behavior, since bone primarily fails under excessive compression rather than tension [28]. In this analysis, positive values represent tensile stresses, while negative values correspond to compressive stresses. The results were presented in tension graphs, with the distribution of tensions and their respective numerical values.

## Results

**Fig. 3:**
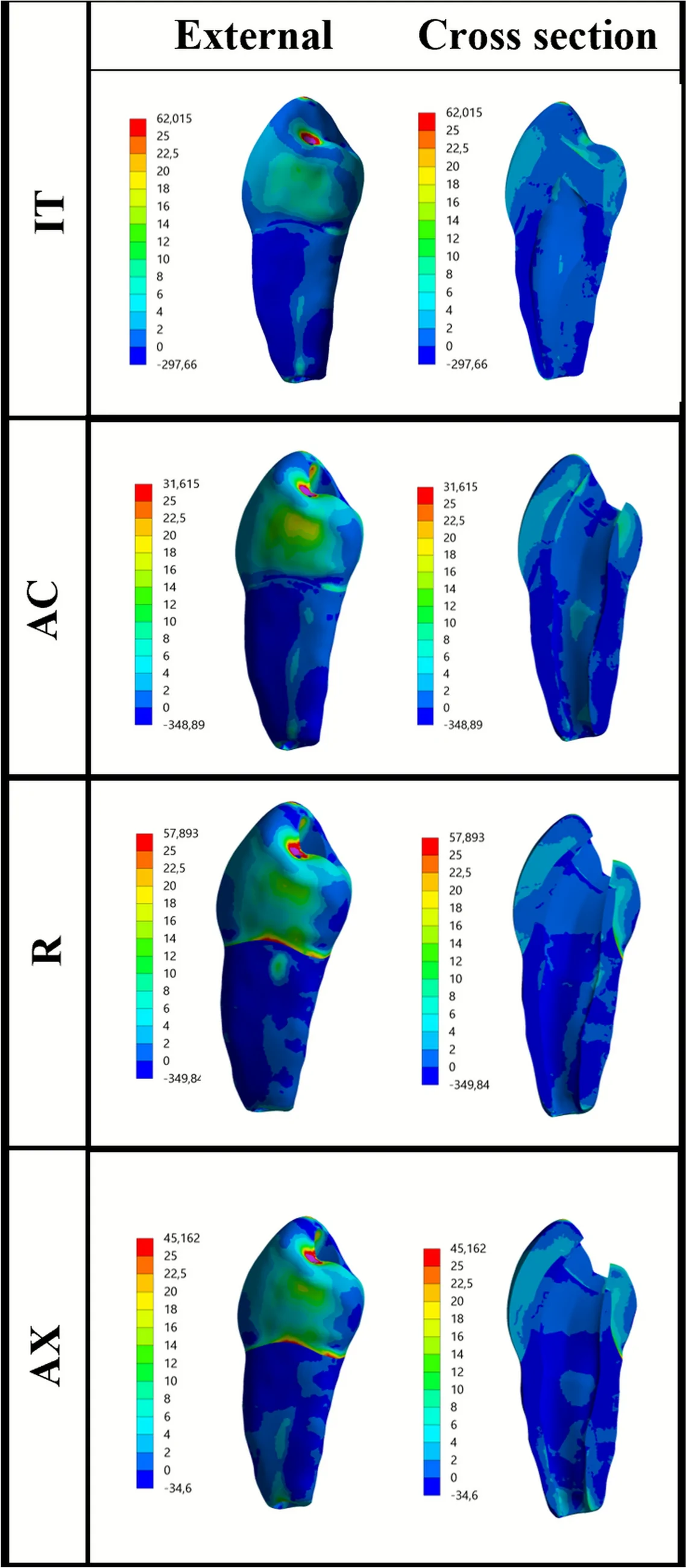
Three-dimensional finite element models of the lower first premolar showing the Maximum Principal Stress distribution for each study group. Note the Maximum Principal Stress distribution in cervical dentin (R and AX) and apical regions (AX). The color scale represents stress intensity in MPa. Legend**:** IT – intact tooth; AC – accessed tooth; R – revascularization; AX – apexification

Figure 3 presents the tensile and compressive stress in immature roots (MPa) through a color map. Areas with tensile stress correspond to positive values, while compressive stress represents negative values.

The stress distribution values in immature lower premolar roots are shown in Table 2. The IT (Intact Tooth) group exhibited the highest tensile stress in the coronal dentin, while the R (Revascularization) and AX (Apexification) groups showed slightly lower values, with the AC (Accessed Tooth) showing lower values than these groups. Instead, a higher compressive stress distribution was observed in the R group, followed by the AC, IT, and AX groups.

**Table 2:** Peak maximum (tensile) and minimum (compressive) principal stress (in MPa) values obtained by finite element analysis for each experimental group and anatomical region. Positive values indicate tensile stress, and negative values indicate compressive stress. PDL stresses were calculated only for the revascularization (R) and apexification (AX) groups, which included simulated periodontal tissues

Groups	Maximum (coronal)(MPa)	Minimum (apical) (MPa)	Maximum (apical) PDL (MPa)	Maximum (buccal) PDL (MPa)	Maximum (lingual) PDL (MPa)
IT	205.73	–364.69	–	–	–
AC	191.31	–348.89	–	–	–
R	203.75	–349.84	–	–	–
R (for alveolar bone)	2.738	–28.53	–	–	–
R (PDL)	203.75	–349.84	7.629	13.034	20.235
AX	203.14	–349.82	–	–	–
AX (for alveolar bone)	3.274	–33.273	–	–	–
AX—PDL	203.14	–349.82	14.105	15.274	21.396

The tensile stress distribution in the color displacement map was observed in the cervical region of the R and AX groups (red areas) and is also seen in the apical region of the AX group, represented by orange (Fig. 3). However, the IT and R groups showed the highest tensile stress distribution when compared to the AC group.

It was possible to observe tensile stress concentration in the distal region (green/yellow area) in the R, AX, and AC groups.

From an external view, the periodontal ligament in the AX group showed higher Minimum Principal Stress values, indicating greater tensile behavior compared to the R group.

**Fig. 4:**
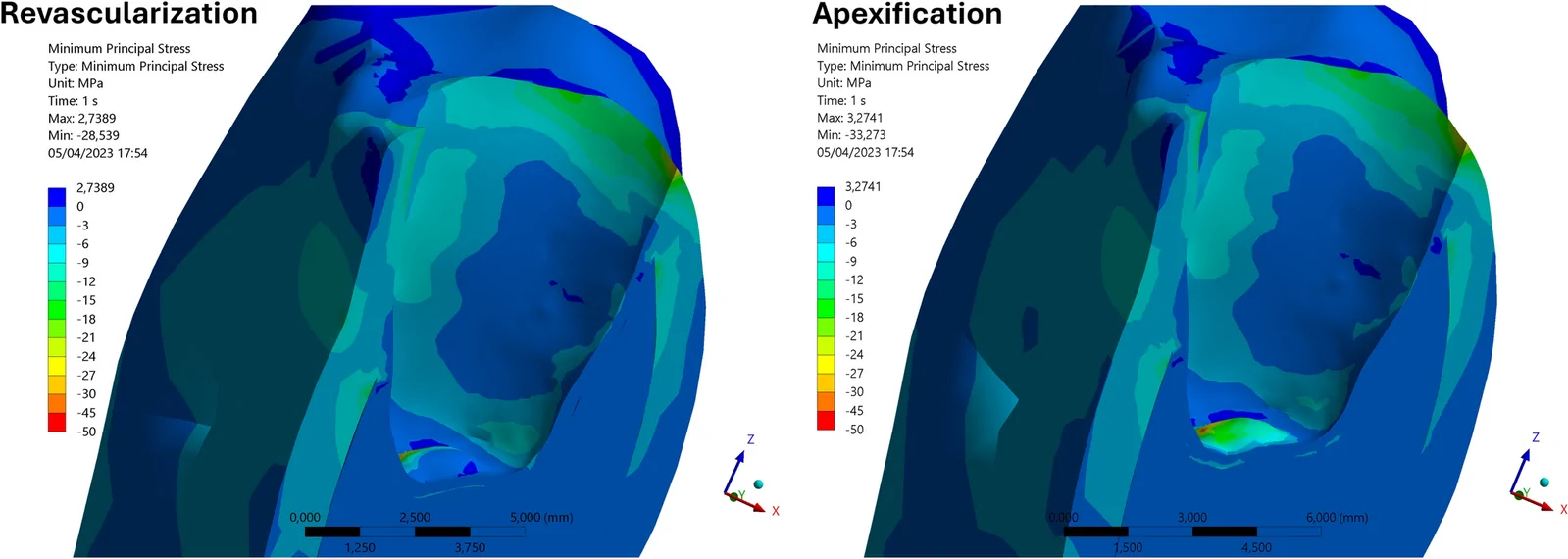
Three-dimensional finite element representation of the alveolar bone of the lower first premolar showing the Minimum Principal Stress distribution for the revascularization and apexification groups. The color scale represents stress intensity in MPa

Figure 4 shows the Minimum Principal Stress (compressive stress) map in the alveolar bone of the R and AX groups. It is possible to observe that a compressive stress concentration is highlighted in the apical zone in the AX group. The R group presented compressive stress values closer to 0 than the AX group (Table 2).

**Fig. 5:**
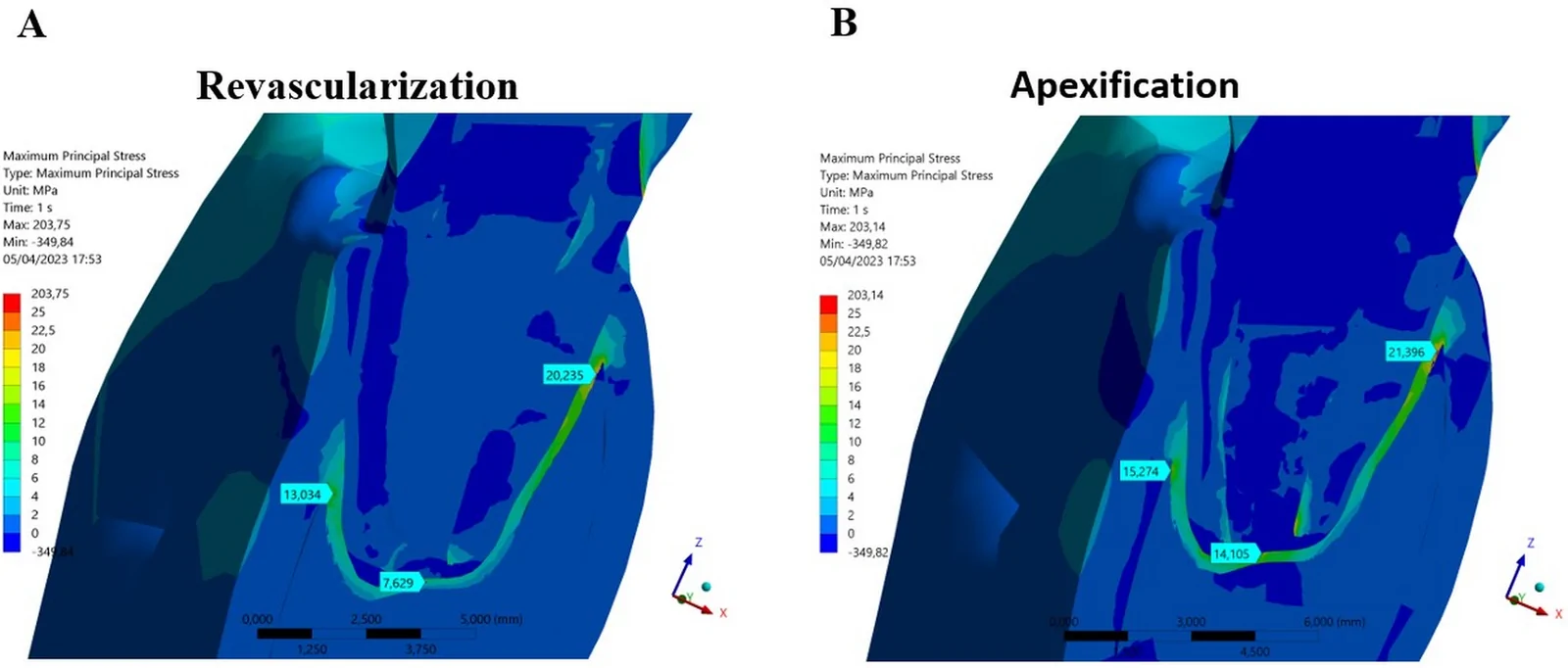
Three-dimensional finite element representation of the periodontal ligament (PDL) of the lower first premolar showing the Maximum Principal stress distribution for the revascularization **A** and apexification **B** groups. The color scale represents stress intensity in MPa

Figure 5 also shows the Maximum Principal Stress distribution of the R and AX groups, but in the periodontal ligament. The revascularization technique showed the lowest tensile stress distribution values in three root zones (buccal, lingual, and apical) when compared to the AX group (Table 2).

## Discussion

This study evaluated the mechanical behavior through FEA analysis of a premolar submitted to revascularization and apexification processes. The revascularization group showed higher tensile stress distribution inside the tooth. In addition, this technique presented the lowest compressive stress distribution value in the alveolar bone and periodontal ligament. Therefore, the tested hypothesis was accepted.

Tensile and compressive stress are different forces that can be analyzed by FEA. Tensile stress is the force that acts on a structure, causing stretching or extension in it. Tensile stress is generally developed in the horizontal direction, while compressive stress causes compression or shortening and is developed in the vertical direction [29]. Tensile stress is represented by positive values, and compressive stress is represented by negative values. Both forces are defined by the ratio between the applied force and the cross-sectional transversal area [30].

The experimental models in this study showed an immediate effect of revascularization and apexification endodontic procedure treatments on immature teeth. Previous studies have already shown that the fracture resistance of roots is directly associated with the remaining tooth structure and dentin amount [31, 32]. Healthy teeth have a sufficient dentin wall to provide adequate resistance to these elements [33]. However, immature teeth submitted to revascularization or apexification present thin root walls due to the non-deposition of secondary dentin. As a result, they become susceptible to fractures [34]. These studies corroborate our results since the IT showed higher tensile stress values when compared to the R and AX groups (Table 2).

The R, AX, and AC groups showed tensile stress distribution in the distal region due to the force application in the distal sulcus occlusal surface. This stress distribution can also be observed on the cervical surface of the AX and R groups (Fig. 3). These groups presented a lower amount of dentin, which is not favorable to its mechanical strength according to the results found by Valera et al. (2004) [34].

The minimum principal stress observed in dentin (–349.84 MPa) is higher than the typical compressive strength reported in the literature [35, 36]. This outcome may be attributed to local numerical stress concentrations at the boundary conditions and the idealized geometric simplifications inherent in the finite element model. In this context, these values represent localized peak stresses rather than realistic clinical magnitudes.

The finite element analysis demonstrated that the revascularization (R) technique resulted in slightly higher tensile stresses within coronal dentin (203.75 MPa) compared to apexification (203.14 MPa) (Table 2). In this way, the revascularization procedure increases stress within the dentin, which may elevate the risk of fracture in structurally compromised teeth [37]. Conversely, revascularization showed markedly lower compressive stresses (for alveolar bone: –28.53 MPa) compared with AX (for alveolar bone: –33.27 MPa), indicating that R may protect supporting tissues and reduce the risk of periradicular damage or resorption, which also can be favorable for long-term tooth support [38]. Clinically, this means that while revascularization may biologically favor continued root development and periradicular tissue preservation, it also increases stress concentration in coronal dentin, potentially elevating the risk of fracture in structurally compromised teeth. Apexification, conversely, may reduce dentin stress but offers less protection to supporting tissues. Within this perspective, revascularization can be preferred for preserving periapical integrity. However, careful case selection and reinforcement strategies (e.g., restorative approaches to support thin dentin walls) should be considered to mitigate the higher dentin stresses.

Some limiting factors in the revascularization technique can be highlighted, such as its technical execution to stimulate bleeding, clot stabilization, and application of a cervical stop [39–42]. However, the revascularization technique has shown high success rates in clinical trials, showing the apexification process as a viable treatment option. The outcome success rate of apexification with MTA and revascularization is shown in the literature to have values of 80.77% and 76.47%, respectively [43].

A study by Ali et al. (2019) [41] showed that bovine incisors treated by the apexification technique with MTA did not affect the mechanical strength when compared to a healthy tooth [44]. On the other hand, Mello et al. (2020) [45] evaluated the mechanical performance of teeth after apexification and revascularization, and they detected no statistically significant difference between these groups [45]. Such discrepancies may stem from differences in experimental design, including tooth type (bovine incisors versus human premolars), analysis methods (finite element simulation versus mechanical testing), and loading configuration. These methodological distinctions may explain why the current findings diverge from previous in vitro studies, highlighting the need for further investigations to validate the mechanical behavior of immature teeth under different treatment protocols.

Silujjai & Linsuwanont [43] reported that the increase in root width development after MTA apexification reached rates of 3.30%, while the revascularization technique showed 13.75%. Moreover, rates of 8.55% were found for apexification of root length development, while 9.51% has been reported for revascularization [46]. These rates were updated by Caleza-Jimenez et al. [4], who reported an increase of 3.36% in dentin thickness and 0.29% in root length for the apexification technique, while revascularization increased dentin thickness by 34.57% and 12.75% for root length [46]. However, the revascularization technique in our study showed higher tensile stress distribution when compared to apexification. On the other hand, apexification showed the furthest compressive stress distribution from critical stress (Table 2).

Although revascularization promotes dentin apposition and increased root thickness, the newly formed tissue may present lower mineralization and reduced elastic modulus compared to mature dentin [47]. Consequently, the mechanical continuity between the old and the newly deposited dentin may be compromised, concentrating stresses along the junction area. Furthermore, the irregular dentin deposition pattern reported in revascularized roots may alter stress distribution, explaining the higher dentin stress observed despite the apparent geometric reinforcement.

Silujjai & Linsuwanont [40] suggest that the content within the root canal may be associated with the root mechanical strength, which corresponds to the different mechanical behavior of the groups in this study [43]. The 5 mm of MTA adopted to the revascularization and apexification models followed the same amount used by Darak et al. (2011) [19]. The literature shows that the use of gutta-percha to fill the root canal may not provide additional mechanical performance in roots after MTA apexification. However, revascularization may improve the development of biological tissues around the periapical root area, thereby favoring the mechanical strength of the roots [48, 49].

The failure criterion adopted for the bone is based on the Minimum Principal Stress. In Fig. 4, the color map highlights the compression regions with reddish and orange tones. Since it is common to associate warmer colors with the most critical results, we chose to invert the color scale to maintain this convention. In this way, warm colors continue to represent the most critical areas. Negative values indicate compressive stress, while positive values correspond to tensile stress. According to Papavasiliou et al. [50], the physiological limit,s for compressive stress of cortical bone are 140 to 170 MPa and 72 to 76 MPa for tensile stress [50]. Our results for the alveolar bone stress distribution (Fig. 4) showed compressive stress from –28.53 to –33.27 MPa and tensile stress distribution values of 2.73 to 3.27 MPa which are between the physiological limits of cortical bone.

It is important to explore how the periodontal ligament (PDL) behaves since this structure is submitted to dynamic loads (tensile and compressive stress) during mastication and orthodontic treatments, for example [51]. Additionally, there is limited data on the behavior of the periodontal ligament (PDL) in revascularization and apexification techniques simulated through FEA, which motivated this study to gain a deeper understanding of its behavior [52]. Tensile stress causes a rupture of collagen fibers, which leads to damage in the periodontal ligament [53]. In this sense, Fig. 5 and Table 2 show that the revascularization technique produces less tensile stress distribution compared to the apexification technique, resulting in less damage to the PDL.

Furthermore, the revascularization technique showed higher mechanical strength. These findings suggest that compared to the healthy group, both revascularization and apexification can be performed without inducing bone resorption, as their stress values remain below the critical thresholds reported for dentin [54] and alveolar bone [55] in the literature.

Within the limitations of this study, the dimensions of the access cavity, along with the volume of dentin and enamel removed during endodontic access preparation, play a critical role in influencing the fracture resistance of the tooth, and it can vary [56]. Moreover, although the adoption of a 10% threshold for mesh convergence has been employed in previous dental FEA studies [57, 58], it is considered less stringent than the conventionally recommended 1–5% variation in peak stress values between successive mesh refinements. The use of a broader tolerance may introduce a higher degree of numerical uncertainty, particularly in regions of elevated stress concentration, such as coronal dentin. In addition, calcifications can occur after revascularization treatment, but they were not simulated in this study due to their high variability [14]. Another limitation of FEA is that it assumes material properties to be homogeneous, linear elastic, and isotropic. While these modeling assumptions are common in FEA studies, they may still affect the clinical translatability of our findings.

The simplification of material properties as isotropic, linearly elastic, and homogeneous can lead to under- or over-estimation of absolute stress values, particularly in tissues such as the PDL and blood clot. Such an assumption disregards their inherently viscoelastic and time-dependent behavior, which could influence local stress and strain distributions. Nevertheless, because the present model simulated a single static loading condition, the use of elastic parameters provides a reasonable first approximation of their short-term mechanical response. In addition, the static loading approach does not reproduce fatigue or dynamic effects that may contribute to long-term failures in vivo. Consequently, although our results reliably capture comparative trends between apexification and revascularization, caution is warranted when interpreting absolute stress magnitudes in a clinical context. Practically, this suggests that while revascularization appears favorable for reducing periradicular (PDL and alveolar bone) stresses, apexification may better preserve dentin integrity. Nonetheless, true clinical outcomes will ultimately depend on biological adaptation, loading variability, and restorative strategies.

Despite these limitations, FEA remains a practical tool, offering reduced computational time and more efficient analysis for simulating and studying the behavior of complex systems that are difficult to replicate in vitro.

The lack of scientific findings comparing the mechanical strength after the revascularization and apexification procedures in immature premolars encouraged our research group to conduct this study. This paper intends to help clinicians better understand the mechanical behavior after performing these techniques. Nevertheless, more studies are needed to complement our findings.

## Conclusion

Within the limitations of this finite element analysis, revascularization and apexification exhibited distinct biomechanical behaviors in immature premolars. Revascularization reduced stress concentration in periradicular tissues, which may favor long-term tooth support. However, it also increased tensile stress within dentin, indicating a higher fracture susceptibility in teeth with thin dentinal walls. In contrast, apexification decreased tensile stress in dentin but led to greater stress concentration in periradicular tissues. Clinically, this suggests a trade-off: while revascularization may better preserve periapical integrity, it requires careful case selection and restorative reinforcement to minimize fracture risk, whereas apexification may offer better short-term dentin preservation at the expense of periradicular protection.

## Data Availability

The datasets used and/or analyzed during the current study are available from the corresponding author on reasonable request.
